# 54. Type I cryoglobulinaemia: vascular occlusion and thrombosis

**DOI:** 10.1093/rap/rky034.017

**Published:** 2018-09-20

**Authors:** Hannah Zacharias, Jasroop Chana, Kieran Sandhu

**Affiliations:** Rheumatology, Bucks Healthcare NHS Trust, Aylesbury, UNITED KINGDOM

**Introduction:** Cryoglobulins are immunoglobulins in the serum that precipitate at temperatures below 37 °C and can be classified into three groups. Type 1 cryoglobulinaemia occurs in approximately 5-25% of cases and develops in the setting of protein secreting monoclonal gammopathies such as a monoclonal gammopathy of undetermined significance (MGUS) or a B-cell lineage malignancy for example multiple myeloma, Waldenström macroglobulinemia, or chronic lymphocytic leukaemia. Cryoglobulinaemia remains asymptomatic in the majority but can lead to immune complex tissue deposition, causing cryoglobulinaemic small and medium vessel vasculitis in up to 15%. Type I cryoglobulinemia classically produces symptoms secondary to vascular occlusion and thrombosis by the cryoprecipitate such as digital ischemia, livedo reticularis, and skin necrosis. Livedo reticularis, vascular occlusion and digital ischaemia are also features of anti-phospholipid syndrome (APLS). It is also noted that there are reports of APLS in the setting of malignancy including myeloproliferative disorders.

**Case description:** We describe the case of a 44 year old female non-smoker, who presented to the vascular surgeons with ischaemic right 2^nd^ and 4^th^ toes and swollen erythematous legs. CT angiogram did not reveal macrovascular occlusion and a subsequent MRA lower limb confirmed there was no significant arterial disease. She had a normal echocardiogram with no evidence of emboli. Due to her history of obstetric APLS she was anticoagulated with warfarin under haematology guidance. This diagnosis was based on 5 first trimester miscarriages following 1 successful pregnancy, one positive lupus anticoagulant and a weakly positive anti-cardiolipin IgG antibody (28 units/ml). She had a second successful pregnancy covered with LMWH. Shortly after discharge, she developed a livedo rash on her lower limbs which rapidly progressed to multiple, excruciatingly painful, punched-out ulcers. She had no additional symptoms and was systemically well. However, her right 2nd and 4th toes were now gangrenous. Investigations at that time showed normal FBC and U+E, ESR 52 and CRP 4.9. Her serology revealed a weakly positive lupus anticoagulant (ratio 1.43), and negative anti-cardiolipin and B2GP1 antibodies. Her ANA, ENA, dsDNA, RF and ANCA were negative. Complement C3 was normal mg/dl and C4 low mg/dl. Interestingly there was a monoclonal IgG band identified with no significant secondary hypogammaglobulinaemia. Urinalysis was negative for casts and proteinuria. She was commenced on prednisolone 20mg for vasculitic ulcers secondary to APLS. She initially improved and the gangrenous toes were surgically amputated. Histology showed severe acute inflammation of the soft tissues, osteomyelitis and small vessel vasculitis. Azathioprine was commenced cautiously at 25mg daily due to a borderline TPMT level. However, her leg ulcers deteriorated reducing prednisolone to 12.5mg daily and she also developed a new purpuric rash on her lower limbs. This progressed to extensive cutaneous necrosis and new right 3rd toe ischaemia. There was no improvement despite 2 pulses of 500mg intravenous methyl prednisolone and iloprost infusion. She underwent amputation of the toe with the histology demonstrating widespread small vessel thrombosis with ischaemic changes but no vasculitic features. Further investigation revealed the presence of cryoglobulins (3% on quantification) in association with an IgG kappa paraprotein of 6g/L with a kappa lambda ratio of 9.92. This lead to a diagnosis of type 1 cryoglobulinaemia. Her bone marrow aspirate showed 3% plasma cells with an abnormal lymphoplasmacytoid infiltrate. There was no excess CD138/CD38 cells and no abnormal B cell clone on bone marrow immunophenotyping. CTCAP did not show any evidence of a solid malignancy. Her hepatitis B and C serology was negative and C4 remained low at 8.3 mg/dl with no additional change in her previous serology. Warfarin was stopped; she was started on treatment dose LMWH and she commenced 60mg prednisolone. She began weekly plasmapheresis and 6 cycles of cyclophosphamide 15mg/kg for cryoglobulinaemic vasculitis. Treatment was complicated by significant wound infection and a skin graft was planned once stable. She also developed new joint synovitis requiring an increase of prednisolone to 40mg daily. Her cryocrit levels continued to rapidly accumulate post plasma exchange. After completing 5 cycles of cyclophosphamide a repeat bone marrow biopsy was performed. This showed 10% plasma cells positive for CD138 and CD56 with kappa light chain restriction and a diagnosis of myeloma was confirmed. She was consented to start treatment with Bortezomib chemotherapy. Unfortunately, she was admitted one week later with sepsis and an acute kidney injury (Creatinine 279 from a baseline of 60). Wound swab from her legs grew an ESBL-producing strain of Escherichia Coli and she was treated with Meropenem. During admission she developed reduced GCS with a dense right sided hemiparesis. Urgent CT brain scan showed a large volume acute parenchymal haemorrhage throughout the left frontal lobe, with intraventricular rupture including the fourth ventricle and generalised oedema. Despite emergency neurosurgical intervention, she deteriorated with another cerebral bleed and passed away.

**Discussion:** In patients with monoclonal (type I) cryoglobulin associated with a lymphoproliferative disorder, the treatment is focused on the underlying malignancy and the risk or presence of complications such as hyperviscosity syndrome. Patients with hyperviscosity may require treatment with plasma exchange. Treatment for vasculitis includes glucocorticoids and cyclophosphamide, Rituximab is effective in mixed cryoglobulinaemia, however in type I the efficacy is less clear. The use of other drugs, including thalidomide, lenalinomide, and bortezomib-based regimens may also be useful. The therapeutic challenge was to control our patient's limb threatening skin manifestions whilst a diagnosis of haematological malignancy was confirmed. The cutaneous lesions were difficult to treat despite multiple therapies and plasmapheresis was not effective in achieving an adequate response. Concurrent infection also proved a challenging factor in giving further immunosuppression. Without a correct diagnosis we could not proceed with the appropriate treatment of the underlying disorder. There is good evidence for the use bortezomib, a proteasome inhibitor that inhibits angiogenesis and production of paraproteins, in treatment in type-1 cryoglobulinemic vasculitis, unfortunately our patient passed away prior to commencing treatment.

**Key Learning Points:** This case highlights the importance of an early diagnosis and treatment. There was approximately 18 months taken from initial presentation to diagnosis with significant implications to patients’ quality of life during the disease process. Clinicians should have a high level of suspicion in those patients presenting with atypical livedo rash and necrosis for type I cryoglobulinemia. Although rare, prompt treatment with plasmapheresis and immunosuppression may be life and limb saving.

**Disclosure: H. Zacharias:** None. **J. Chana:** None. **K. Sandhu:** None.

